# A multilayer biomaterial for osteochondral regeneration shows superiority vs microfractures for the treatment of osteochondral lesions in a multicentre randomized trial at 2 years

**DOI:** 10.1007/s00167-017-4707-3

**Published:** 2017-09-14

**Authors:** Elizaveta Kon, Giuseppe Filardo, Mats Brittberg, Maurizio Busacca, Vincenzo Condello, Lars Engebretsen, Stefan Marlovits, Philipp Niemeyer, Patrik Platzer, Michael Posthumus, Peter Verdonk, Renè Verdonk, Jan Victor, Willem van der Merwe, Wojciech Widuchowski, Claudio Zorzi, Maurilio Marcacci

**Affiliations:** 1 Humanitas University Department of Biomedical Sciences - Humanitas Clinical and Research Center, Milan, Italy; 2 NABI Laboratory, Rizzoli Orthopaedic Institute, Via Di Barbiano 1/10, 40136 Bologna, Italy; 3 Department of Orthopaedics, Cartilaginous research unit, Goteborg University, Kungsbacka Hospital, Kungsbacka, Sweden; 4 Radiology, Rizzoli Orthopaedic Institute, Bologna, Italy; 5 Dipartimento di Ortopedia, Ospedale Sacro Cuore Don Calabria di Negrar, Verona, Italy; 6 Department of orthopaedic surgery, Ullevål Hospital, Oslo University, Oslo, Norway; 7 Ordinationszentrum Döbling, Vienna, Austria; 8 Department of orthopaedic surgery and traumatology, Freiburg University Hospital, Freiburg Im Breisgau, Germany; 9 Department of traumatology, Medical University of Vienna, Vienna, Austria; 10 Division of Exercise Science and Sports Medicine, Faculty of Health Sciences, The University of Cape Town, Cape Town, South Africa; 11 Antwerp Orthopaedic Center, Monica Hospitals, Stevenslei, Deurne, Belgium; 12 Université Libre de Bruxelles, Brussels, Belgium; 13 Department of orthopaedic surgery, Ghent University Hospital, Ghent, Belgium; 14 Sport Science Orthopaedic Clinic, Sport Science Institute of South Africa Newlands, Cape Town, South Africa; 15 Wojewódzki Szpital Chirurgii Urazowej, II Oddział Urazowo-Ortopedyczny, Piekary Śląskie, Polen

**Keywords:** Osteochondral, Cartilage, Scaffold, Knee, Bone marrow stimulation

## Abstract

**Purpose:**

The increasing awareness on the role of subchondral bone in the etiopathology of articular surface lesions led to the development of osteochondral scaffolds. While safety and promising results have been suggested, there are no trials proving the real potential of the osteochondral regenerative approach. Aim was to assess the benefit provided by a nanostructured collagen–hydroxyapatite (coll-HA) multilayer scaffold for the treatment of chondral and osteochondral knee lesions.

**Methods:**

In this multicentre randomized controlled clinical trial, 100 patients affected by symptomatic chondral and osteochondral lesions were treated and evaluated for up to 2 years (51 study group and 49 control group). A biomimetic coll-HA scaffold was studied, and bone marrow stimulation (BMS) was used as reference intervention. Primary efficacy measurement was IKDC subjective score at 2 years. Secondary efficacy measurements were: KOOS, IKDC Knee Examination Form, Tegner and VAS Pain scores evaluated at 6, 12 and 24 months. Tissue regeneration was evaluated with MRI MOCART scoring system at 6, 12 and 24 months. An external independent agency was involved to ensure data correctness and objectiveness.

**Results:**

A statistically significant improvement of all clinical scores was obtained from basal evaluation to 2-year follow-up in both groups, although no overall statistically significant differences were detected between the two treatments. Conversely, the subgroup of patients affected by deep osteochondral lesions (i.e. Outerbridge grade IV and OCD) showed a statistically significant better IKDC subjective outcome (+12.4 points, *p* = 0.036) in the coll-HA group. Statistically significant better results were also found for another challenging group: sport active patients (+16.0, *p* = 0.027). Severe adverse events related to treatment were documented only in three patients in the coll-HA group and in one in the BMS group. The MOCART score showed no statistical difference between the two groups.

**Conclusions:**

This study highlighted the safety and potential of a biomimetic implant. While no statistically significant differences were found compared to BMS for chondral lesions, this procedure can be considered a suitable option for the treatment of osteochondral lesions.

**Level of evidence:**

I.

**Electronic supplementary material:**

The online version of this article (doi:10.1007/s00167-017-4707-3) contains supplementary material, which is available to authorized users.

## Introduction

Chondral and osteochondral lesions are debilitating conditions that, if not properly treated, may lead to the development of osteoarthritis, with a high impact on patients and society, both in terms of healthcare and workforce loss [14, 15]. The greater emphasis on physical activity in all age groups is responsible for the growing incidence of these lesions, and concomitantly, patient expectations about function recovery have risen as well. Thus, in the attempt to fulfil patients’ expectations and successfully treat this pathology, several techniques have been developed over the years [26].

The available surgical options range from simple strategies such as microfracture (MF) or subchondral drilling to the more ambitious regenerative approaches. MF is a bone marrow stimulation (BMS) technique aimed at recruiting bone marrow cells by creating a communication between cartilage lesions and subchondral bone, thus allowing stem cells to migrate to the fibrin clot of the defect and form a fibrocartilaginous repair tissue [36]. Regenerative procedures aim at recreating a hyaline-like tissue as similar as possible to the physiological one, and they are emerging as a potential therapeutic option also in cases of large lesions, where other procedures are less indicated [1, 32]. Several materials have been developed in the attempt to meet the requirements of cartilage regeneration. The rationale for using a scaffold is to offer a temporary 3-dimensional structure of biodegradable polymers to mimic cartilage architecture and favour cell growth [24]. However, despite the increasing number of publications every year confirming good outcomes also at midterm evaluations [1], the superiority of this technique versus BMS has not been clearly proven, and MF is still considered the gold standard that sets the reference point to measure the potential of new procedures [8].

The increasing awareness on the role of subchondral bone in the etiopathology of articular surface lesions may explain the limits of the current regenerative procedures, which have been developed to target only the chondral layer [9, 24]. Subchondral bone may be involved in the pathological process not only primarily, such as in osteochondritis dissecans (OCD), osteonecrosis and trauma, but also secondarily in degenerative cartilage lesions. In fact, even focal chondral defects, if left untreated, may increase in size over time and cause changes of the underlying subchondral bone [25, 33]. Thus, a surgical approach for both cartilage and bone reconstruction that would address articular surface lesions by restoring the properties of the entire osteochondral unit should be considered. Among the many different multilayer scaffolds developed to reproduce both bone and cartilage [35], only a few have been investigated in clinical studies [22]. While safety and promising results have been suggested [21, 22], there are no trials proving the real potential of the osteochondral regenerative approach compared to the gold standard in clinical practice.

The aim of this study is to assess the benefit provided by a nanostructured collagen-hydroxyapatite (coll-HA) multilayer scaffold in a randomized controlled trial. The hypothesis is that this comparative evaluation will demonstrate safety and a superior clinical benefit of this osteochondral scaffold.

## Materials and methods

The study was performed in compliance with the protocol and in accordance with the Declaration of Helsinki, the International Conference of Harmonization Guidelines for Good Clinical Practice, standards from International Organization for Standardization (ISO) and valid international and national regulations. Patients referring to specialized orthopaedic centres in the following countries were included: Italy, Sweden, Belgium, Switzerland, Austria, Germany, Norway, Poland, South Africa. From 2011 to 2013, 145 patients affected by chondral and osteochondral knee lesions were screened; 124 patients were randomized (safety population).

**Fig. 1:**
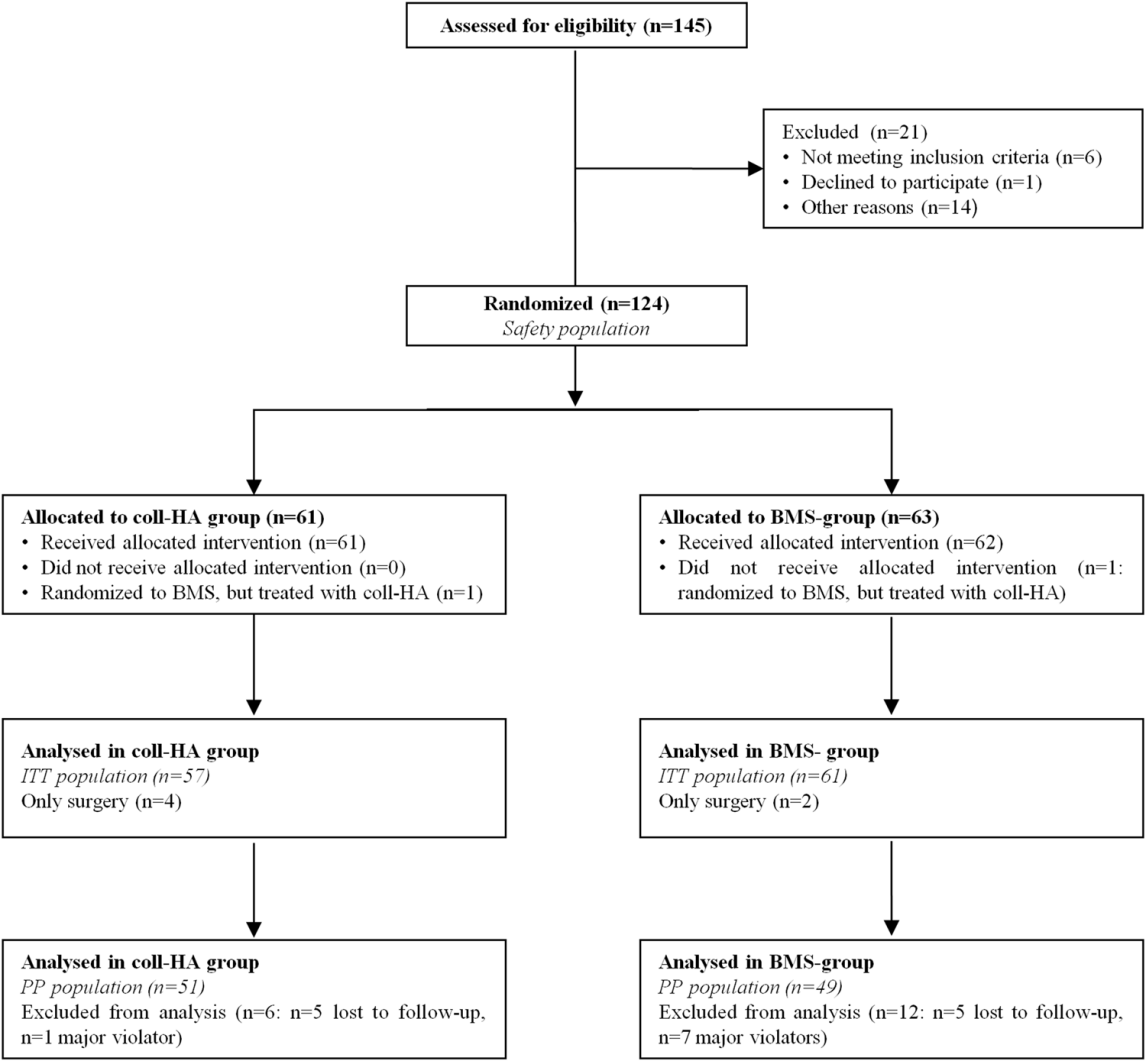
Flow chart: diagram of the patients eligible, randomized, ITT population and PP population

The selected ITT population consisted of 118 patients, while the PP population resulted in 100 patients, due to protocol violators and dropouts (Fig. 1). Study and BMS group presented, respectively, a mean age of 34.0 ± 10.9 and 35.2 ± 10.2 years old; a male/female ratio of 36/15 and 31/18; a body mass index of 25.6 ± 3.3 and 25.2 ± 3.2 and a mean defect size of 3.4 ± 1.5 and 3.5 ± 1.6 cm^2^. Further demographics data have been reported in detail in Table 1. These patients were evaluated for up to 2 years of follow-up with both clinical and imaging examination.

**Table 1:** Demographics of the coll-HA and BMS groups

		Coll-HA	BMS
Patients		51	49
Age (years)	Mean ± SD	34.0 ± 10.9	35.2 ± 10.2
Gender *n* (%)	Male	36 (70.6%)	31 (63.3%)
	Female	15 (29.4%)	18 (36.7%)
BMI (kg/m^2^)	Mean ± SD	25.6 ± 3.3	25.2 ± 3.2
Lesion dimension (cm^2^)	Mean ± SD	3.4 ± 1.5	3.5 ± 1.6
Lesion localization *n* (%)	Condyle	37 (72.6%)	23 (47.0%)
	Trochlea	2 (3.9%)	6 (12.2%)
	Patella	12 (23.5%)	20 (40.8%)
Aetiology *n* (%)	Microtraumatic/degenerative	20 (39.2%)	24 (49%)
	OCD	15 (29.4%)	12 (24.5%)
	Traumatic	13 (25.5%)	12 (24.5%)
	Other	3 (5.9%)	1 (2%)
Associated surgery *n* (%)		19 (37.3%)	14 (28.6%)
Previous surgery *n* (%)		27 (52.9%)	23 (46.9%)
Pre-surgery activity level *n* (%)	Non-active	35 (68.6%)	38 (77.5%)
	Sport active	16 (31.4%)	11 (22.5%)

Patients were randomly allocated to either the scaffold or BMS treatment with a *ratio* of 1:1. The randomization list was prepared using the algorithm of Moses Oakford, with allocation blocks of variable sizes and stratified by orthopaedic centres. In order to minimize possible selection biases, the randomization code was kept in a sealed individual patient envelope, which could be opened only just before surgery.

### Treatments

**Fig. 2:**
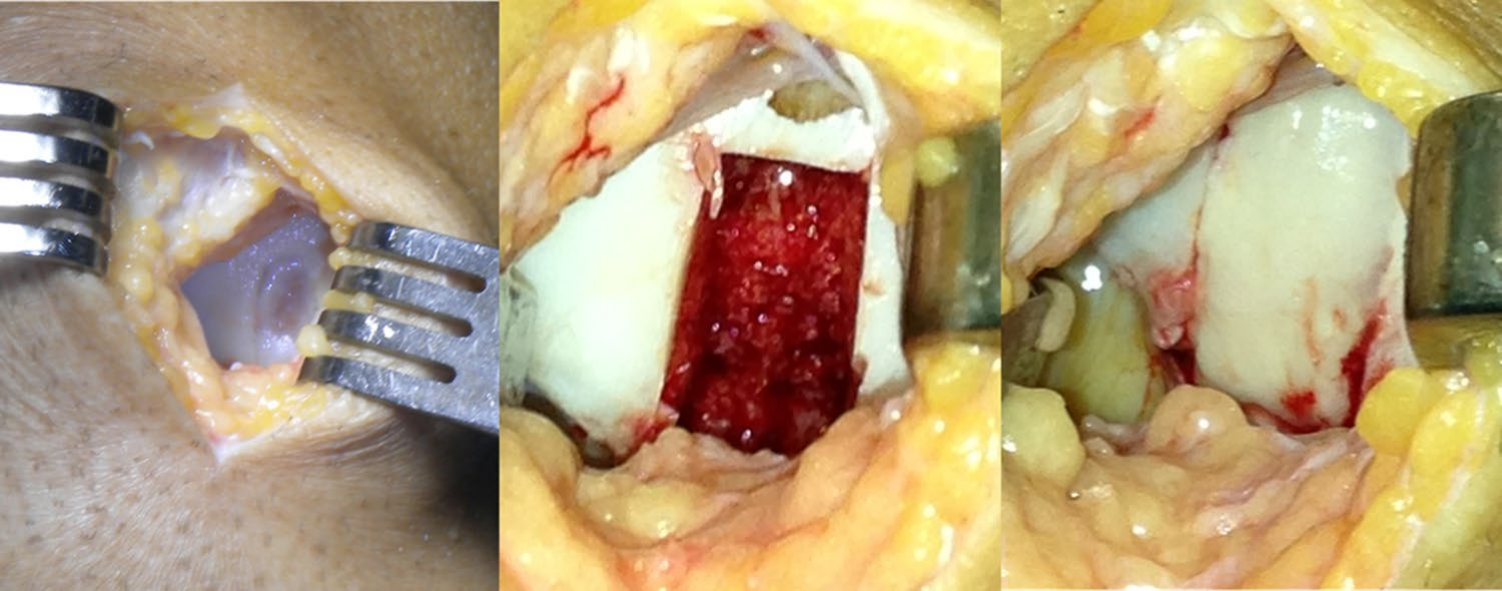
Osteochondral scaffold implantation for a traumatic focal medial femoral condyle lesion of 2 cm^2^ in a 30-year-old man

MaioRegen (Fin-Ceramica Faenza S.p.A., Italy) is a bioceramic composite scaffold, developed to promote the processes of tissue regeneration in case of severe and large chondral (grade III and IV according to Outerbridge classification) and osteochondral lesions, which would otherwise be difficult to treat. The design of this 6-mm-thick scaffold mimics the three-dimensional natural structure of both cartilage and subchondral bone layers: the cartilaginous upper layer is smooth on the surface and consists entirely of Type I equine collagen; the intermediate layer is made of a combination of Type I collagen (60%) and magnesium-enriched HA (Mg-HA; 40%), while the lower layer consists of a mineralized blend of Type I collagen (30%) and Mg-HA (70%). The surgical procedure was performed with the patient in supine position with a pneumatic tourniquet placed on the proximal thigh. An arthrotomic approach with a medial or lateral parapatellar arthrotomy was used to expose the lesions. The defect was then prepared by removing the sclerotic subchondral bone to obtain stable shoulders to house the scaffold, which was implanted by press fit according to the previously described technique [17] (Fig. 2). After implantation, management of post-operative pain allowed for early mobilization starting on the second post-operative day to favour swelling resolution, promote defect healing and prevent adhesions. Early isometric and isotonic exercises and electrical neuromuscular stimulation (NMES) were indicated and could be started at patient discharge. By the fourth week, weight touchdown with crutches was allowed, and the patient could then move progressively towards full weight bearing. Swimming and cycling were allowed 1 month after surgery, low active functional training after 4–6 months and joint impact activities after 1 year, following previous literature indications [17].

### Reference treatment

BMS techniques, such as subchondral drilling or MF, are based on the arthroscopic perforation of the subchondral bone plate at the bottom of the cartilage lesion, allowing stem cells from the bone marrow to repopulate and fill the defect with a repair tissue. These methods differ in terms of penetration in the subchondral bone plate, with MF being the less invasive approach and subchondral drilling entailing deeper holes.

Thus, MF was used for smaller lesions, according to the literature indications and with standard Steadman awls [26, 29], while for lesions larger than 4 cm^2^ or with a higher damage of the subchondral bone, such as OCD, subchondral drilling with a Kirschner wire (1.0–1.2 mm in diameter, with penetration left to the surgeon judgment in order to reach the proper depth and ensure enough bleeding and access to progenitor cells) was chosen to ensure the highest chances of success for patients randomized to the BMS approach.

### Outcome measures

Efficacy was evaluated according to the following widely accepted outcome measurements. Primary criteria: IKDC Subjective Knee Evaluation Form 2000 score 2 years after surgery, as proposed by the ICRS (International Cartilage Repair Society) [16]. Secondary criteria: patient functional improvement through KOOS, IKDC Knee Examination Form 2000, Tegner scores [7, 16, 38] evaluated at each follow-up visit, together with patient pain assessment (VAS Pain). Tissue regeneration was evaluated at each follow-up visit with 1.5-T MRI. All post-operative MRIs were centrally and blindly evaluated by senior musculoskeletal radiologists by applying the most commonly accepted score for the evaluation of cartilage treatments (MOCART scoring system) [28], modified to assess the subchondral bone health state (supplementary material). For the detailed description of the sequences used and the parameters evaluated see supplementary material. Each MRI was evaluated by two radiologists; in case of discrepancy of the result, a third radiologist was involved.

Safety was evaluated focusing on the number and type of adverse events after surgery (pain, movement restriction, infection, inflammation, device expulsion or mobilization, etc.). Failure was defined as the need for reintervention on the same defect based on the persistence or recurrence of symptoms.

An external independent agency (Contract Research Organization, CRO) was involved to ensure data correctness and objectiveness of the study results. In particular, planning and investigation were performed according to the Standard Operating Procedures of CROMSOURCE (Verona, Italy) to ensure the protection of the rights and the integrity of the subjects, adequate and correct conduct of all study procedures, data collection, documentation and data verification.

The trial, coordinated by the Rizzoli Orthopaedic Institute (Bologna, Italy), was approved by the local hospital and regional ethics committees, and written informed consent of all patients was obtained before enrolment (Prot. 0020721 Trial registration: NCT01282034).

### Statistical analysis

A blinded statistician carried out the analysis.

In order to minimize the influence of the protocol violators on the results and the possible bias of focusing on ITT populations in surgical studies, by ignoring patient noncompliance with the original assignment, it was deemed opportune to focus further analysis on the PP population. This population, free of carryover effects (dropouts) and of major protocol violations (pre-specified before unblinding), is more reliable as it consists of patients who adhered perfectly to the clinical trial instructions as stipulated in the protocol. Consequently, results on the PP population would more likely exhibit the effects of treatment according to the underlying scientific model. The comparative analysis is based on an ANCOVA model with change from baseline to 2-year follow-up in IKDC Subjective Knee Evaluation score as dependent variable, treatment, centre and location as fixed effects and baseline as covariate. Results of the primary analysis are presented as a point estimate, a 95% confidence interval and an associated *p* value for the adjusted mean difference between coll-HA and BMS groups, for the PP population.

Sample size: the study was designed to demonstrate the superiority of coll-HA scaffold compared to BMS techniques. The null hypothesis for the treatment comparison was that there would be no difference between coll-HA (test) and BMS techniques (reference). The alternative hypothesis was that there would be a difference. A two-sided t test with *α* = 0.05 was used to test this hypothesis. Based on the evidence from the pilot study [17], and assuming seven points as the minimum acceptable difference in the modification of the IKDC Subjective Knee Evaluation Score between coll-HA and BMS therapy, the sample size for every group in the study was estimated in 67 patients, in order to ensure a power equal to 90% and a first type error alpha of 5%. From 2011 to 2013, 145 patients were screened and 124 randomized. Due to protracted enrolment period and after an interim analysis, it was decided to interrupt the study once 118 treated patients were reached (ITT population). Excluding major violators and dropouts, the PP population consisted in 100 patients who were treated and evaluated for up to 2 years of follow-up (51 study group and 49 BMS group) (Fig. 1).

## Results

A statistically significant improvement of all clinical scores was obtained from baseline to 2-year follow-up in both treatment groups.

### Coll-HA group

**Fig. 3:**
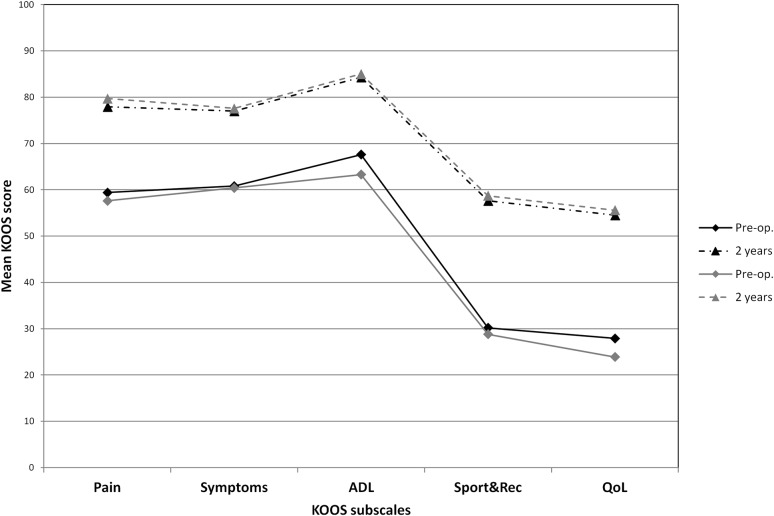
Change from baseline to 2-year follow-up of KOOS profile (black: coll-HA and grey: BMS)

The IKDC subjective VAS Pain, Tegner and KOOS scores, as well as the percentage of patients with “Normal” or “Nearly normal” knees, improved as detailed in Tables 2, 3 and Fig. 3. In this group, one centre had a statistically significant worse performance when compared to others (*p* < 0.05). Nevertheless, it was decided to maintain the centre in the statistical analyses since no errors were observed in the management of the study.

**Table 2:** Outcome evaluations pre-operatively, at 1 and 2 years of follow-up

	Coll-HA			BMS				
	Pre-op. (mean ± SD)/ median (range)	1 year (mean ± SD)/ median (range)	2 years (mean ± SD)/median (range)	Pre-op. (mean ± SD)/median (range)	1 year (mean ± SD)/median (range)	2 years (mean ± SD)/median (range)	Adjusted mean difference between treatments	*p* value*
Subj. IKDC^a^								
Total	43.2 ± 16.6	60.7 ± 17.3	66.7 ± 21.0	41.1 ± 15.9	61.8 ± 18.0	63.6 ± 18.2	−0.482	n.s.
Deep osteochondral lesions	42.4 ± 17.5	66.8 ± 15.3	77.8 ± 15.6	38.9 ± 12.9	60.0 ± 16.6	64.3 ± 18.1	**12.437**	**0.036**
Sport active patients	50.1 ± 15.4	68.2 ± 19.0	76.3 ± 20.4	44.5 ± 20.5	63.1 ± 16.4	64.2 ± 15.9	**15.946**	**0.027**
OCD	50.3 ± 18.8	71.3 ± 16.3	76.7 ± 18.8	36.4 ± 9.7	59.5 ± 14.7	61.9 ± 18.0	11.946	n.s.
VAS–Pain^a^	50.1 ± 26.7	23.8 ± 20.8	26.5 ± 27.5	53.1 ± 22.7	29.2 ± 23.2	23.2 ± 20.9	6.553	n.s.
Tegner^b^	3.0 (0.0; 7.0)	4.0 (2.0; 7.0)	4.0 (1.0; 9.0)	3.0 (0.0; 9.0)	4.0 (1.0; 9.0)	4.0 (2.0; 8.0)	0.139	n.s.

*p* value* on adjusted mean difference between treatments (coll-HA–BMS)

Bold values indicate statistical significance

The values are given as ^a^ the mean with standard deviation or ^b^ median and range in parentheses

**Table 3:** Final evaluation from IKDC Knee Examination Form from pre-operatively to 1 and 2 years of follow-up

	Coll-HA			BMS		
	Pre-op. (%)	1 year (%)	2 years (%)	Pre-op. (%)	1 year (%)	2 years (%)
Normal	49.0	76.5	80.4	36.7	87.8	73.5
Nearly normal	33.3	17.6	13.7	42.9	10.2	16.3
Abnormal	11.8	3.9	3.9	14.3	0.0	6.1
Severely abnormal	3.9	2.0	0.0	4.1	0.0	0.0
Missing/ND	2.0	0.0	2.0	2.0	2.0	4.1

### BMS group

The IKDC subjective, VAS Pain, Tegner and KOOS scores improved, as detailed in Table 2 and Fig. 3. There was no statistically significant difference from baseline to 2-year follow-up in the percentage of patients with “Normal” or “Nearly normal” outcome according to the IKDC Knee Examination Form changed (Table 3).

### Comparison between coll-HA group and BMS group

The comparative analysis showed no statistically significant difference in IKDC subjective score at 2-year follow-up between the two treatment groups. Similarly, the overall outcome measured with VAS Pain, KOOS, IKDC Knee Examination Form and Tegner scores did not show any statistically significant difference.

### Imaging evaluation

**Fig. 4:**
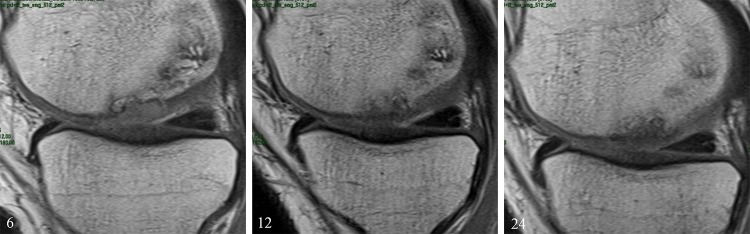
Magnetic resonance imaging evaluation of a coll-HA implant in the medial femoral condyle, showing ongoing osteochondral remodelling at 6 months, 1 and 2 years of follow-up

With regard to the imaging evaluation of tissue regeneration, the MOCART score showed no statistical difference in the MRI score between the two groups. In the coll-HA population, a reduction in the effusion was observed up to 2 years and features consistent with ongoing bone remodelling were found. (Details are reported in Table 4, Fig. 4.)

**Table 4:** MOCART score

	Coll-HA			BMS		
	6 months	1 year	2 years	6 months	1 year	2 years
	*n* = 45 pts (%)	*n* = 49 pts (%)	*n* = 51 pts (%)	*n* = 43 pts (%)	*n* = 45 pts (%)	*n* = 44 pts (%)
Degree of defect repair and filling of the defect						
Complete	53.3	40.8	49.0	39.5	55.6	65.9
Hypertrophy	33.3	36.7	37.3	18.6	13.3	18.2
Incomplete, >50% of the adjacent cartilage	8.9	20.4	13.7	34.9	31.1	11.4
Incomplete, <50% of the adjacent cartilage	2.2	2.0	0.0	4.7	0.0	2.3
Subchondral bone exposed	2.2	0.0	0.0	2.3	0.0	2.3
Integration to border zone						
Complete	40.0	61.2	60.8	53.5	60.0	70.5
Incomplete, demarcating border visible	48.9	30.6	37.3	32.6	35.6	22.7
Incomplete, defect visible, <50%	8.9	8.2	2.0	7.0	4.4	4.5
Incomplete, defect visible, >50%	2.2	0.0	0.0	7.0	0.0	2.3
Surface of the repair tissue						
Surface intact	48.9	49.0	56.9	46.5	62.2	56.8
Surface damaged, < 50%	42.2	46.9	41.2	44.2	35.6	40.9
Surface damaged, > 50%	8.9	4.1	2.0	9.3	2.2	2.3
Structure of the repair tissue						
Homogeneous	8.9	16.3	13.7	55.8	46.7	45.5
Inhomogeneous or cleft formation	91.1	83.7	86.3	44.2	53.3	54.5
Signal intensity of the repair tissue–dual T2-FSE						
Isointense	26.7	24.5	41.2	55.8	64.4	61.4
Moderately hyper-/hypo-intense	66.7	73.5	56.9	39.5	35.6	36.4
Markedly hyper-/hypo-intense	6.7	2.0	2.0	4.7	0.0	2.3
Signal intensity of the repair tissue—DP FAT-SAT						
Isointense	26.7	18.4	43.1	41.9	48.9	47.7
Moderately hyper-/hypo-intense	60.0	75.5	52.9	51.2	48.9	47.7
Markedly hyper-/hypo-intense	13.3	6.1	3.9	7.0	2.2	4.5
Subchondral bone						
Intact	44.4	24.5	11.8	41.9	42.2	25.0
Minimal changes	51.1	69.4	70.6	51.2	48.9	65.9
Marked changes	4.4	6.1	17.6	7.0	8.9	9.1
Oedema						
Absent	35.6	42.9	43.1	30.2	35.6	52.3
Slightly hyperintense and/or small oedema	62.2	51.0	49.0	65.1	57.8	40.9
Markedly hyperintense and/or extensive oedema	2.2	6.1	7.8	4.7	6.7	6.8
Adhesions						
No	100	100	100	100	100	100
Effusion						
No	28.9	40.8	62.7	67.4	71.1	68.2
Yes	71.1	59.2	37.3	32.6	28.9	31.8

### Adverse events

Safety was evaluated focusing on number and type of adverse events after surgery in all patients randomized and treated (124 patients). Severe treatment-related adverse events were documented in 3 patients in the coll-HA group and in 1 patient in the BMS group. Two failures were detected in the study group. (Details of all safety-related events are reported in Table 5.)

**Table 5:** Safety evaluation of coll-HA and BMS groups

	Coll-HA	BMS
Safety population	62 patients	62 patients
No. of adverse events related: Minor early post-operation symptoms Inflammation Joint adhesions Persistent pain Loose body Joint instability	13 (total) 8 3 1 1 0 0	4 (total) 3 0 0 0 0 1
No. of serious adverse events related: Joint adhesions Persistent pain Loose body	3 (total) 2 1 0	1 (total) 0 0 1
No. of failures	2	0

### Predicting factors influencing the final outcome

In the group treated with coll-HA, factors predicting a favourable or a negative outcome were: Outerbridge grade IV (factor predicting positive outcome: *p* < 0.05) and concomitant ACL treatment (factor predicting negative outcome: *p* < 0.05), while other parameters such as age, sex and lesion size did not influence the final outcome. No predicting factors were present in the BMS group.

**Fig. 5:**
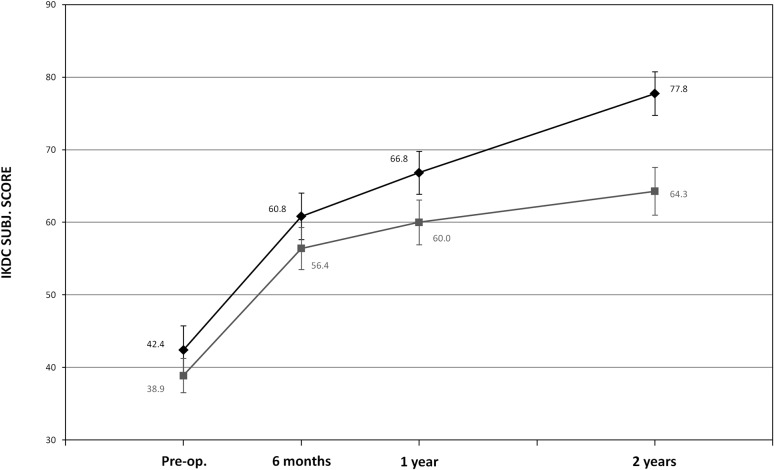
Change from baseline to 2-year follow-up of IKDC Subjective Knee Evaluation score in deep osteochondral lesions subgroup (*black*: coll-HA and *grey*: BMS)

Further analyses were performed to identify whether specific patients’ subgroups (chosen according to factors found to influence coll-HA results and to scientific literature) may present more or less benefit compared to the applied treatments. Accordingly, patients affected by deep osteochondral lesions (patients suffering from osteochondral lesions severely involving subchondral bone, i.e. Outerbridge grade IV and OCD) with no associated ACL surgery were identified as group of interest. This subgroup included 27 patients treated with coll-HA and 30 with BMS; the analysis showed statistically higher values in terms of adjusted means of changes from baseline to 2 years: 33.6 (95% CI) for coll-HA vs 21.2 (95% CI) for the BMS group at 2 years of follow-up. Change from baseline to 2 years in IKDC Subjective Knee Evaluation score showed a statistically significant (*p* = 0.036) adjusted mean difference of +12.4 points in favour of coll-HA (Table 2, Fig. 5).

**Fig. 6:**
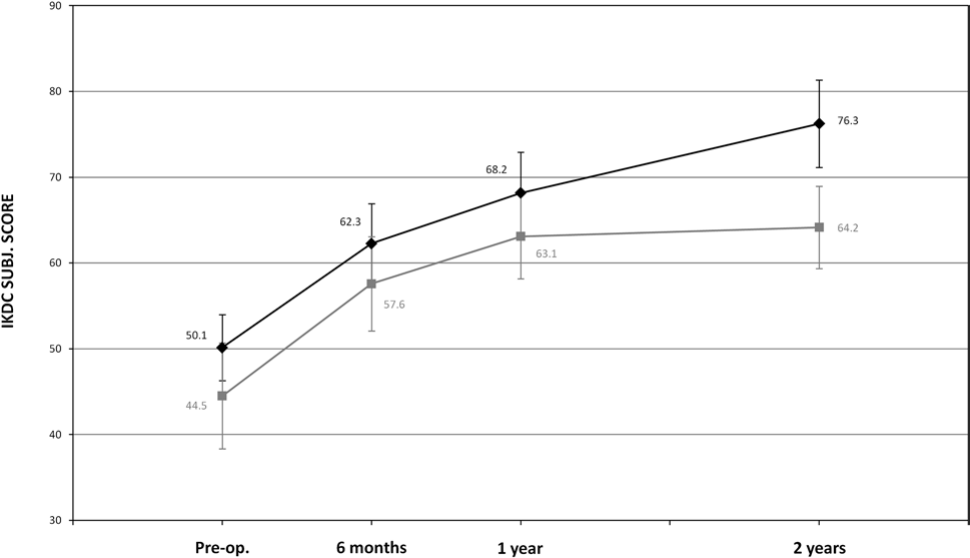
Change from baseline to 2-year follow-up of IKDC Subjective Knee Evaluation score in sport active patients’ subgroup (*black*: coll-HA and *grey*: BMS)

A superior outcome was also found for the sport active patients’ subgroup (16 patients in the coll-HA group and 11 in the BMS group), with a statistically higher (*p* = 0.027) adjusted IKDC subjective mean difference of +16.0 points in favour of coll-HA at 2-year follow-up (Table 2, Fig. 6).

**Fig. 7:**
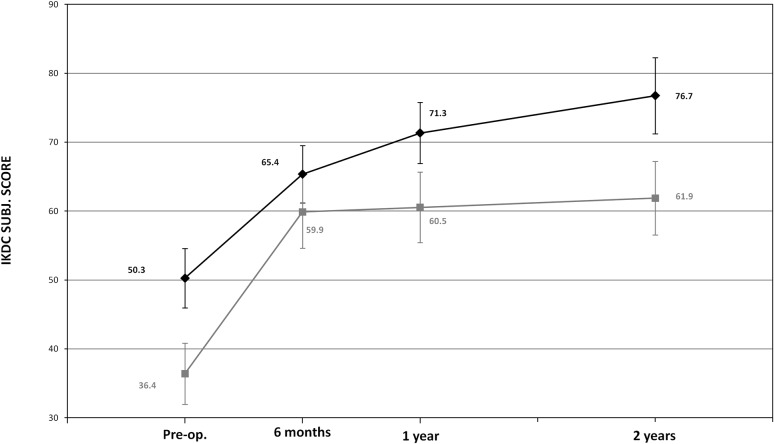
Change from baseline to 2-year follow-up of IKDC Subjective Knee Evaluation score in OCD subgroup (*black*: coll-HA and *grey*: BMS)

Finally, in the subgroup of patients affected by OCD (15 patients treated with coll-HA and 12 with BMS), there was a clinically relevant improvement in patients treated with coll-HA. Change from baseline to 2-year follow-up in IKDC Subjective Knee Evaluation score showed an adjusted mean difference of +12.0 points between the two treatments, which is clinically meaningful although not statistically significant in this study (*p* = 0.144) (Table 2, Fig. 7).

## Discussion

The main finding of this study is that comparable results were found in the overall population, whereas the coll-HA osteochondral scaffold offered significant clinical better results compared to BMS in the treatment of osteochondral lesions. BMS techniques confirm to be a treatment option for purely chondral lesions, but offer worse results for more complex lesions, where the biomimetic scaffold, designed to address also the subchondral area, showed to be a more suitable therapeutic solution.

The reason for this selected superiority lies in the rational of the treatments themselves, and the mechanisms leading to these different results reflect previous literature findings. Smaller chondral lesions are those benefiting more from BMS, whose potential to achieve good results over time finds the main limitation in the lesion size [13]. In fact, the repair tissue response is often fibrous and cannot address successfully larger defects [31]. This, together with the damage produced by the technique at the subchondral bone level [30], may explain the inferior results obtained with BMS in more complex lesions. On the other hand, the composite scaffold has been developed to mimic the biochemical and biophysical properties of different layers of the native osteochondral structure [37] to favour the repair of the entire osteochondral unit [34]. The properties of this scaffold have been shown to produce good results in preclinical models [23, 34]. The scaffold ability to induce an in situ repair through cells coming from the surrounding bone marrow in the animal model [18] allowed to introduce it in the clinical practice as a cell-free approach.

Safety and preliminary experiences with human application have been documented in case series published by different groups. Delcogliano et al. obtained an encouraging outcome at 24-month follow-up on 19 patients affected by large-sized defects [5], confirmed by Verdonk et al. evaluating at the same follow-up 38 patients affected by osteochondral lesions [39]. These positive findings were also confirmed by Berruto et al. evaluating 49 patients in a multicentre study which supported the potential of this scaffold also for the treatment of large osteochondral lesions [2]. This biomimetic scaffold was also tested as salvage procedure in young patients for the treatment of unicompartmental OA, where subchondral cysts, oedema and stiffening may be addressed by the osteochondral scaffold, an attempt to provide an alternative solution to metal resurfacing that showed promising short-term results [12, 27]. Finally, some encouraging findings have also been documented in the only available comparative evaluation versus a chondral scaffold for the treatment of complex lesions, with a better outcome in the osteochondral treatment group [11].

This multicentre study confirms safety and potential of the osteochondral scaffold, showing for the first time, with a randomized study design, the improvement provided compared to BMS when targeting complex osteochondral knee lesions. This benefit is supported by the clinical outcome documented through a subjective functional evaluation. More adverse events were reported in the study group. However, most of them were minor post-op symptoms that spontaneously resolved within 45 days. The higher rate of adverse events may be explained by the different invasiveness of coll-HA and BMS surgical approaches, i.e. arthrotomic versus arthroscopic, which have been shown to affect the early recovery of the patients [19]. Summarizing, this trial shows how to address the entire osteochondral unit and the usefulness of this biomimetic implant.

Nonetheless, the imaging evaluation confirms some concerns previously risen in the literature on the slow restoration of the subchondral bone area. Even though the slow bone mineralization did not correlate with a lower clinical outcome, their significance and impact in terms of tissue regeneration and clinical outcome remains to be determined. Suboptimal imaging findings, not correlating with clinical results, have been shown at short-term evaluation by Verdonk et al. and Christensen et al. in particular in terms of subchondral bone formation [2, 4]. On the other hand, Brix et al. underlined a limited cartilage quality but a successful osteoconduction [3]. While further studies need to explore the clinical impact of the observed alterations, the only available midterm evaluation of this osteochondral scaffold shows stable clinical results, as well as a significant MRI improvement between 2 and 5 years of follow-up [20]. This may suggest that the maturation required by an osteochondral scaffold may be longer than what expected for chondral scaffolds (as confirmed by this RCT). This is probably due to the greater complexity of a scaffold aiming at restoring the entire osteochondral unit and also by the bigger size of the tissues integrating and regenerating, being not just a thin layer but rather a thick layer of articular surface. Moreover, controversial findings may be also partially explained by MRI itself, which in a recent meta-analysis was shown to fail a correlation in the majority of the parameters observed, with only 28% of the studies presenting a correlation between MRI and treatment outcomes [6]. Since the scores available for cartilage studies were designed for the evaluation of cartilage treatments, it is easy to understand that it is even more difficult to expect a correlation in case of an osteochondral treatment evaluation. Therefore, caution should be recommended when interpreting MRI findings and greater importance should be placed on the patient clinical evaluation, until more reliable imaging instruments will be available.

This study presents some limitations. The number of originally planned patients has not been reached, which could have hampered the possibility to detect further significant differences. Nonetheless, the study groups allowed to document the superiority of the osteochondral scaffold in deep osteochondral lesions, which is in line with previous preliminary literature findings [11] and what one may expect from an osteochondral biomimetic implant. Patients were rather heterogeneous in terms of age, lesion dimension, location and aetiology, and one-third required combined surgeries. This, on the one hand, may represent a confounding factor, but it also allows to have a study population more representative of the general population commonly requiring cartilage treatments, which increases the generalizability of these study findings. Despite its heterogeneity, a tendency to treat small lesions was observed in this study, and this might have entailed a bias in favour of the BMS group, as it is possible that coll-HA yields more favourable results in bigger lesions, while MF is more suitable for smaller lesions. Thus, the superiority found for complex lesions regardless of this limitation further confirms the potential of this scaffold. The study presents also other limitations: the 2-year follow-up does not allow to detect the outcome decline of BMS shown by previous literature [8, 13], neither to understand the clinical meaning and evolution of the abnormal MRI findings observed in the scaffold implant area, which warrants further evaluation at longer follow-up. To this regard, the only study currently available at midterm follow-up showed stable clinical outcome and a trend towards slow imaging improvement, even though MRI abnormalities persisted even at 5-year follow-up [20]. The understanding of the tissue and clinical evolution over time is therefore particularly important because it could allow to better identify both treatment potential and most suitable indications. In fact, many questions remain still open, athletes tend to present good early results with several procedures, thus the benefit shown in this study should be confirmed by longer-term evaluations, and it will be important to understand whether bone repair, which may explain the better results in osteochondral lesions, is coupled by sufficient cartilage repair in order to allow good and stable results overtime in young active patients.

Future studies should therefore focus on osteochondral healing mechanisms and on further improvement of the regenerative potential of this scaffold. To this regard, technical advancements have been already identified, such as the possibility to enhance the scaffold fixation with the use of fibrin glue or to use thinner scaffolds for shallower lesions, thus prompting the possibility to further increase the outcome of the scaffold [10]. Moreover, while preclinical evaluations did not show any benefit in chondrocyte seeding [18], further studies should explore whether other augmentation strategies could improve the osteochondral regenerative potential either with chondro/osteoinductive molecules or taking advantages from the combination with other cell sources to promote a faster healing with a higher quality tissue and in the end better clinical results.

While the clinical applicability of the aforementioned augmentation procedures may be affected by economic and regulatory requirements, this study highlighted the safety and potential of the biomimetic coll-HA implant showing, for the first time with a randomized trial, its superiority versus BMS approach in the treatment of osteochondral defects. Thus, this procedure can be considered as an option to address the damaged articular surface when subchondral bone is also involved, with the advantage of being an off-the-shelf product requiring a one-step surgery and offering good results for the treatment of the osteochondral surface.

## Conclusions

This RCT showed safety and an overall positive clinical outcome provided by this coll-HA biomaterial. While comparable results were found in the overall population, the osteochondral scaffold offered significant clinical better results compared to BMS in the treatment of osteochondral lesions. BMS techniques confirm to be a treatment option for purely chondral lesions, but offer worse results for more complex lesions, where the biomimetic scaffold, designed to address also the subchondral area, showed to be a more suitable therapeutic solution.

## Electronic supplementary material

Below is the link to the electronic supplementary material.

Supplementary material 1 (DOCX 15 kb)
