# Supplementary material for: A multilayer biomaterial for osteochondral regeneration shows superiority vs microfractures for the treatment of osteochondral lesions in a multicentre randomized trial at 2 years
Source: Knee Surg Sports Traumatol Arthrosc. 2017 Sep 14;26(9):2704–15. doi: 10.1007/s00167-017-4707-3 (PMC6105149; doi:10.1007/s00167-017-4707-3)
Supplement: Supplementary file 1 — Supplementary material 1 (DOCX 15 kb) [file 167_2017_4707_MOESM1_ESM.docx]

**MRI acquisition protocol**

Each MRI evaluation was carried out on a MRI 1.5 Tesla super conducting magnet with a dedicate knee coil.

Studies of the femoral condyles should be performed with the following technique:

- Sagittal and coronal FSE proton density weighted with fat saturation (TR: 3200 ms; TE: 22 ms; ET: 8; FOV 160 mm; matrix: 512 x 256; slice thickness: 3 mm; gap 0.5 mm; NEX: 2; number of slices: 24),
- Sagittal Dual FSE (TR: 2800-3200 ms; TE: shortest ms and 100-120 ms; ET: 8; FOV 160 mm; matrix: 512 x 256; slice thickness: 3mm; gap 0.5 mm; NEX: 2; number of slices: 24),
- Coronal 3D-gradient echo sequence with fat-suppression (TR: 40 ms; TE: 5 ms; flip angle 45°; FOV 160 mm; matrix: 256 x 256; slice thickness: 2.0 mm; NEX: 3; number of slices: 60-70),
- Coronal FSE proton density weighted with fat saturation (TR: 3200 ms; TE: 22 ms; ET: 8; FOV 160 mm; matrix: 512 x 256; slice thickness: 4 mm; gap 0.5 mm; NEX: 2; number of slices:18).

The following sequences should be applied for the patella and trochlea:

- Sagittal and coronal FSE proton density weighted with fat saturation (TR: 3200 ms; TE: 22 ms; ET: 8; FOV 160 mm; matrix: 512 x 256; slice thickness: 3 mm; gap 0.5 mm; NEX: 2; number of slices: 24),
- Sagittal Dual FSE (TR: 2800-3200 ms; TE: shortest ms and 100-120 ms; ET: 8; FOV 160 mm; matrix: 512 x 256; slice thickness: 3mm; gap 0.5 mm; NEX: 2; number of slices: 24),
- Axial 3D-gradient echo sequence with fat-suppression (TR: 40 ms; TE: 5 ms; flip angle 45°; FOV 160 mm; matrix: 256 x 256; slice thickness: 2.0 mm; NEX: 3; 24 slices),
- Axial FSE proton density weighted with fat saturation (TR: 3200 ms; TE: 22 ms; ET: 8; FOV 160 mm; matrix: 512 x 256; slice thickness: 3 mm; gap 0.5 mm; NEX: 2; number of slices: 18).

**MOCART score**

For the description and evaluation of the treated lesions the Magnetic Resonance Observation of Cartilage Repair Tissue scoring system (MOCART) has been employed. The MOCART score is composed by nine items to describe the whole appearance of the treated lesion, the morphology and signal intensity of the repair tissue compared to the adjacent native one. For the specific application in this study, some items included in the MOCART score were modified to assess the subchondral bone health state.

In the MOCART score the repair grade of the lesion is considered complete when the repair tissue appears as thick as the adjacent native tissue, with a complete integration of the margins, and a smooth articular surface that reproduces the original articular contour with no adhesions and an intact subchondral bone plate and marrow. The signal intensity of the repair tissue is separately determined in fast spin-echo (dual T2-FSE) and fat-suppressed gradient-echo (3DGE-FS) sequences and a complete repair is graded as isointense, if it appears as intense as the adjacent native cartilage. According to the classification system, only marginal modifications are performed to obtain reproducible results and clear definitions. Thus, the variable synovitis is modified into the variable effusion, where the appearance of effusion is defined when the accumulation of fluid in the synovial joint increases more than 1cm in any section of the knee.
